# The prognosis of recurrent low-grade endometrial stromal sarcoma: a retrospective cohort study

**DOI:** 10.1186/s13023-021-01802-8

**Published:** 2021-04-07

**Authors:** Qianwen Dai, Baolin Xu, Huanwen Wu, Yan You, Ming Wu, Lei Li

**Affiliations:** 1 Department of Obstetrics and Gynecology, Peking Union Medical College Hospital, Shuaifuyuan No. 1, Dongcheng District, Beijing, 100730 China; 2 Department of Obstetrics and Gynecology, the Second People’s Hospital of Jingdezhen, Jingdezhen, 333099 China; 3 Department of Pathology, Peking Union Medical College Hospital, Beijing, 100730 China

**Keywords:** Low-grade endometrial stromal sarcoma, Fertility-sparing treatment, Ovarian preservation, Surgery, Recurrence, Survival outcomes

## Abstract

**Background:**

The prognosis of recurrent low-grade endometrial stromal sarcoma (LGESS) is little known. This study was to investigate the survival outcomes of a cohort of patients with recurrent LGESS.

**Methods:**

Patients with primary LGESS diagnosed and treated for first recurrence confirmed by histology in the study center from February 2012 to June 2019 were retrospectively included. The progression-free interval (PFI) after the last treatment for first recurrence and overall survival (OS) since the diagnosis of first recurrence, which were followed up to June 1, 2020, were compared between groups of various therapy modalities.

**Results:**

Fifty-six patients were included, and 43 patients (76.8%) had definite follow-up outcomes. The 5-year PFI and OS rates were 30.0% (95% confidence interval [95% CI] 29.2–30.8) and 75.0% (68.0–82.0), respectively. In univariate analysis, only fertility-sparing treatment, ovarian preservation and surgical treatment had a significant impact on the PFI (hazard ratio [HR] 4.5, 3.1, and 0.2; 95% CI 1.5–13.1, 1.3–7.3, and 0.1–0.7; and *p* = 0.006, 0.009 and 0.006, respectively), but no factor was found to be associated with increased mortality risk. After adjusted with hormone treatment or chemotherapy, surgical treatment had significant effectiveness on OS (HR 0.3 and 0.3, 95% CI 0.1–1.0 and 0.1–1.0, *p* = 0.045 and 0.049, respectively). None of the patients with fertility-sparing treatment had successful conception, and all experienced repeated relapse.

**Conclusion:**

For patients with recurrent LGESS, fertility-sparing treatment or ovarian preservation should not be provided. Surgery is the treatment of choice, and hormone treatment and/or chemotherapy was effective for the survival benefits of surgical treatment.

**Supplementary Information:**

The online version contains supplementary material available at 10.1186/s13023-021-01802-8.

## Background

Low-grade endometrial stromal sarcoma (LGESS) is a malignant tumor composed of cells resembling stromal cells of the proliferative-phase endometrium, displaying permeative, infiltrative growth into the myometrium and/or Iymphovascular spaces [1]. LGESS represents < 1% of all uterine malignancies but is the second most common uterine malignant mesenchymal tumor with a favorable prognosis [2, 3]. The five-year disease-specific survival rates are 90% for stage I or II disease and 50% for stage III or IV disease [4, 5]. Due to its indolent course, few studies have reported the prognosis of recurrent LGESS. In a cohort of 42 patients, incomplete surgery and no adjuvant treatment in ESS were associated with poor DFS. Furthermore, resection of recurrent disease is associated with a survival advantage [6], and hormonal treatment for measurable residual or recurrent low-grade ESS has a high response rate [7]. Because of their efficacy and minimal adverse effects, letrozole [8, 9] and medroxyprogesterone [10, 11] have been used to treat patients with recurrent or residual LGESS that is difficult to resect surgically. Patients seem to benefit from surgical removal of metastatic lesions, especially pulmonary lesions, followed by progestin therapy [12]. However, these reports had limited sample sizes of patients with recurrent LGESS and restricted effective decision-making with recurrent patients with LGESS.

In this retrospective cohort study, we enrolled all LGESS cases with first recurrence who were diagnosed and treated in the study center to explore the prognostic characteristics and relevant risk factors. The primary objectives were to analyze the progression-free interval (PFI) after the last treatment for first recurrence and overall survival (OS) since the diagnosis of first recurrence. Since fertility-sparing and ovarian preservation surgeries have been shown to be associated with an increased risk of recurrence in LGESS patients [13, 14], we paid special attention to the impact of these issues on the PFI after recurrence.

## Methods

### Ethical approval

The Institutional Review Board from the study center approved this retrospective study (No. SK-1289). All procedures performed in the study involving human participants were in accordance with the ethical standards of the institutional and National Research Committee and with the 1964 *Declaration of Helsinki* and its later amendments or comparable ethical standards.

### Study design

This was a retrospective cohort study. Patients with primary LGESS diagnosed and treated for first recurrence in the study center from February 2012 to June 2019 were included. Follow-up of fertility and oncologic outcomes was carried out up to June 1, 2020. The primary and recurrent pathological diagnoses were reviewed and confirmed by two pathologists (HW and YY). Patients were excluded if they had an ambiguous or a misdiagnosis of LGESS, had no histological evidence of recurrence, or did not accept surgical treatment for the primary disease. Clinicopathological information, including age at diagnosis of recurrence, symptoms at recurrence, and treatment modalities for recurrence, was collected by reviewing case reports. Special attention was given to fertility-sparing treatment and ovarian preservation in the treatment of recurrence.

### Interventions and measures

Patients accepted treatment with or without surgical interventions. According to surgical entities, patients were divided into groups with or without fertility-sparing surgery and groups with or without ovarian preservation. In this study, fertility-sparing surgery denotes preservation of the uterus and at least one ovary. In this study, patients with fertility-sparing surgery also had records of regular menstruation after all relevant treatments. Ovarian preservation denotes preservation of at least one ovary with or without an intact uterus. Data on surgical routes and residual lesions from surgical records were also collected from the case and pathology reports. The same attention was given to the chemotherapy regimens and courses, hormone therapy regimens and courses, and radiotherapy.

Oncologic outcomes consisted of PFI after the last treatment for first recurrence and OS since the diagnosis of first recurrence, which were followed up until June 1, 2020. All recurrences were confirmed via histological diagnosis by biopsy and/or repeated surgeries. All deaths were confirmed by certification of death.

### Statistics

Comparisons of continuous variables were conducted with parametric methods if assumptions of a normal distribution were confirmed. Nonnormally distributed variables and categorical data involving specific clinicopathological characteristics were compared between the different groups by using nonparametric tests. Survival curves were generated with the Kaplan–Meier method, and proportional hazards models were used to estimate the hazard ratios (HRs) and 95% confidence intervals (95% CIs) to assess the effects of treatment modalities on PFI and OS. Unless otherwise stated, all analyses were performed with a two-sided significance level of 0.05 and conducted with the use of the software Statistical Product and Service Solutions (SPSS) Statistics 20.0 (IBM Corporation, Armonk, NY, USA).

## Results

### Patient characteristics

**Fig. 1:**
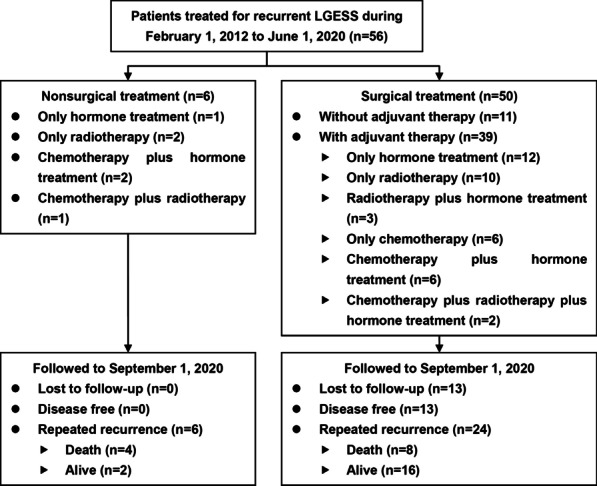
Flow diagram of the study. LGESS, low-grade endometrial stromal sarcoma

The flow diagram is presented in Fig. 1. In total, 56 patients with repeated LGESS were included in the study. The baseline characteristics of the patients are summarized in Table 1. The pathological evaluation suggested most patients had mitotic activity < 5 per 10 high power field (80.4%), or with positive expression of estrogen receptor (ER) (91.1%) and progesterone receptor (PR) (89.3%). The median DFS before recurrence and the median age at diagnosis were 34.7 (range 5–188) months and 43.3 (17–70) years, respectively. Twenty-three patients (41.1%) had metastasis beyond the pelvic cavity or had symptomatic recurrence. Eleven (19.6%), 44 (78.6%) and 1 (1.8%) patients were diagnosed by physical examination, imaging evaluation, and accident findings during cesarean section, respectively. Among 44 cases diagnosed by imaging methods, sonography, computed tomography, magnetic resonance imaging and positron emission tomography were primarily performed in 22 (50.0%), 12 (27.3%), 6 (13.6%) and 4 (9.1%) patients, respectively. Among 23 patients with symptomatic recurrence, abdominal pain or a mass was the most common complaint (12 patients [52.2%]), followed by vaginal bleeding or a mass (6 [26.1%]) and backache (3 [13.0%]). The other two patients complained of ileus and severe fatigue.

**Table 1:** Epidemiological characteristics, treatment and follow-up of enrolled patients

	Values
Extrauterine LGESS, n (%)	9 (16.1)
Mitotic activity < 5 per 10 high power field, n (%)	45 (80.4)
Immunohistochemical staining, n (%)	
Positive estrogen receptor	51 (91.1)
Positive progesterone receptor	50 (89.3)
DFS after first treatment (months), median (range)	34.7 (5–188)
Ages at diagnosis of recurrence (years), median (range)	43.3 (17–70)
Extrapelvic recurrence, n (%)	23 (41.1)
Symptomatic recurrence, n (%)	23 (41.1)
Repeated surgeries for first recurrence, n (%)	50 (89.3)
With residual lesions, n/n (%)	3/50 (6.0)
Chemotherapy for recurrence, n (%)	17 (30.4)
Radiotherapy for recurrence, n (%)	18 (32.1)
Hormone treatment for recurrence, n (%)	26 (46.4)
Fertility sparing	
Fertility sparing after first treatment, n (%)	9 (16.1)
Fertility sparing after first recurrence, n (%)	6 (10.7)
Ovarian preservation	
Ovarian preservation after first treatment, n (%)	35 (62.5)
Ovarian preservation after first recurrence, n (%)	9 (16.1)
Loss to follow-up, n (%)	13 (23.2)
Repeated recurrence, n/n (%)	30/43 (69.8)
PFI after last treatment for first recurrence (months), median (range)	18.3 (1–121)
Death, n/n (%)	12/43 (27.9)
OS after diagnosis of first recurrence (months), median (range)	43.6 (3–349)

DFS, disease-free survival; PFI, progression-free interval; LGESS, low-grade endometrial stromal sarcoma

After recurrence, six patients refused repeated surgery and accepted only GnRHa treatment (1 case), only radiotherapy (2 cases), only chemotherapy (1 case), or medroxyprogesterone plus chemotherapy (2 cases). Among the 50 patients who underwent repeated surgeries, 3 had residual lesions. Among 16 and 32 patients with fertility-sparing surgery and ovarian preservation at first treatment, 5 and 6 accepted repeated fertility-sparing surgery and ovarian preservation treatment, respectively. Chemotherapy, radiotherapy and hormone treatment were utilized by 17 (30.4%), 18 (32.1%) and 26 (46.4%) patients, respectively.

### Survival outcomes

Up to June 1, 2020, 13 patients (23.2%) were lost to follow-up after they finished treatment for recurrence. Among the remaining 43 patients, 30 (69.8%) experienced repeated recurrence, and 12 (27.9%) patients died, with a median PFI and OS of 18.3 (range 1–121) and 43.6 (3–349) months, respectively. The 5-year PFI and OS rates were 30.0% (95% CI 29.2–30.8) and 75% (68–82), respectively, and the 10-year OS rate was 36% (16–56).

**Fig. 2:**
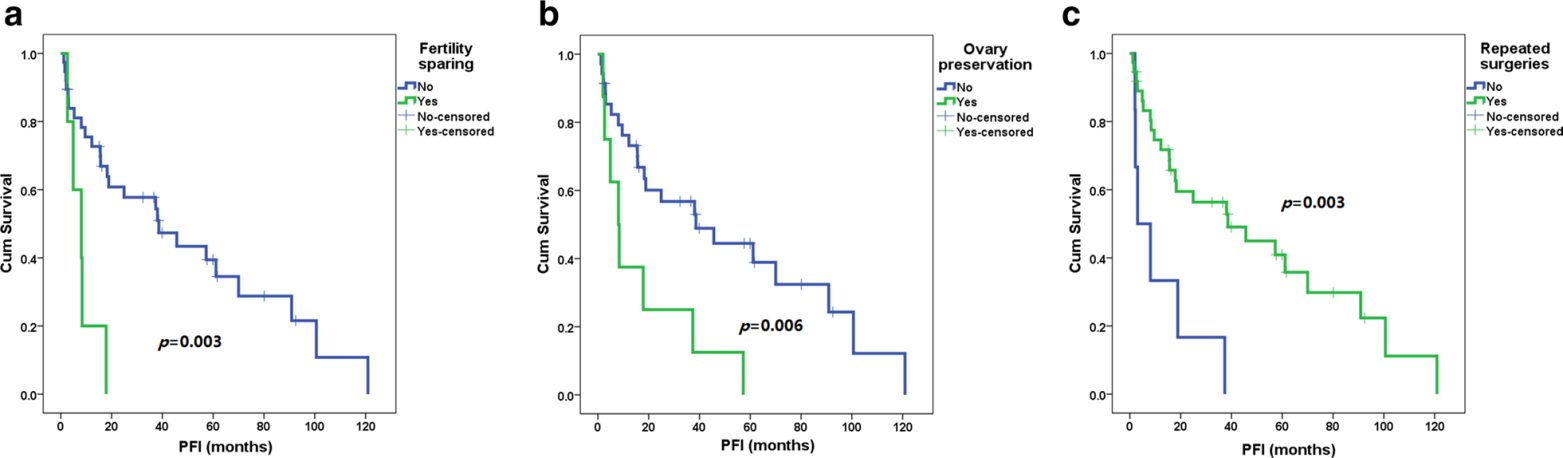
Progression-free interval (PFI) after first recurrence of patients with and without fertility-sparing surgery (**a**), or with and without ovarian preservation (**b**), or with and without repeated surgeries for recurrence (**c**) by Kaplan–Meier analysis

**Fig. 3:**
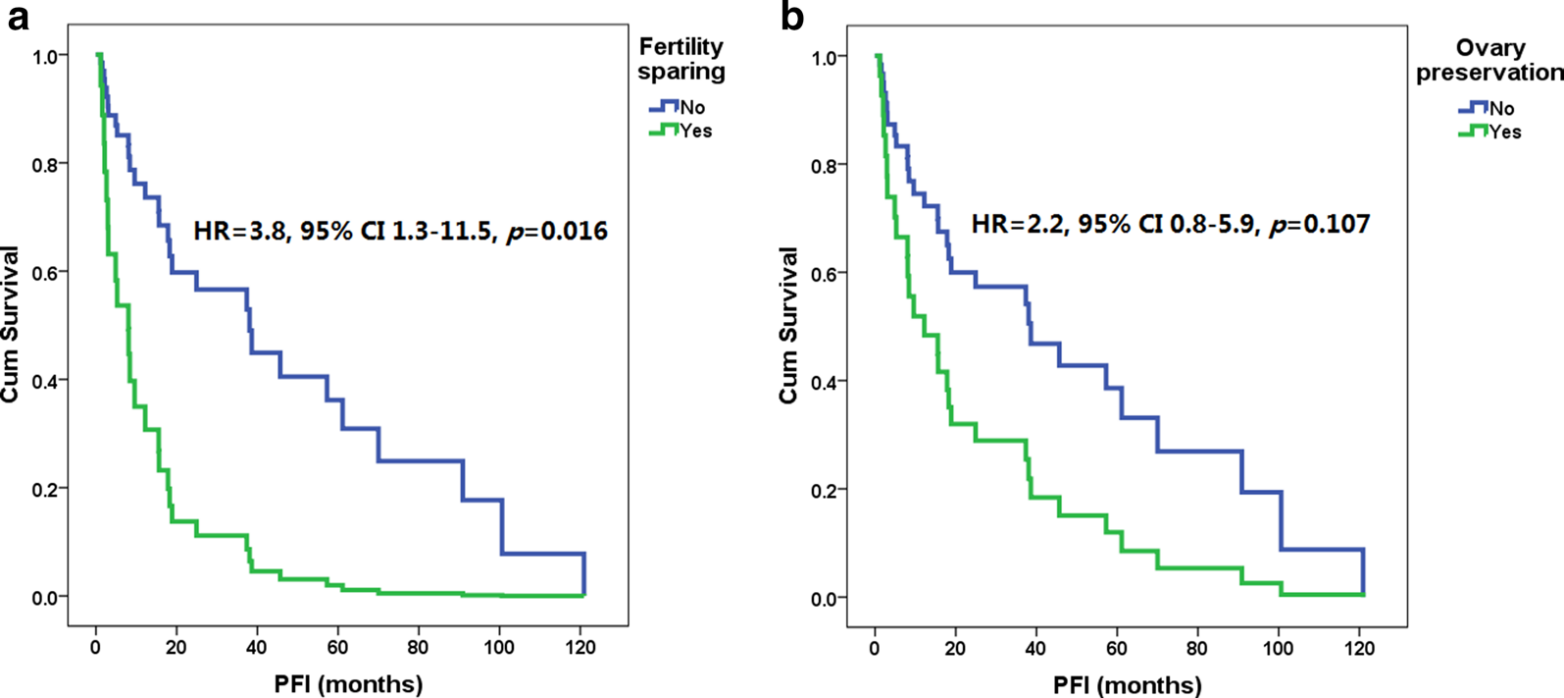
Progression-free interval (PFI) in patients with and without fertility-sparing surgery (**a**) and in patients with and without ovarian preservation (**b**). *95% CI* 95% confidence interval, *HR* hazard ratio

As shown in Fig. 2, in the Kaplan–Meier analysis, fertility-sparing surgery, ovarian preservation and surgical treatment had a significant impact on the PFI after the last treatment for first recurrence (HR 4.5, 3.1, and 0.2, 95% CI 1.5–13.1, 1.3–7.3, and 0.1–0.7, *p* = 0.006, 0.009 and 0.006, respectively). The median PFIs of patients with and without fertility-sparing surgery were 8 (range 3–18) and 29 (1–121) months, the median PFIs with and without ovarian preservation were 8 (2–57) and 25 (1–121) months, and the median PFIs with surgical and nonsurgical treatment were 25 (1–121) and 6 (2–37) months, respectively. As shown in Fig. 3, after adjusted by repeated surgeries, fertility-sparing treatment still resulted in a higher risk of recurrence (HR 3.8, 95% CI 1.3–11.5, *p* = 0.016), but ovarian preservation had no significant impact on OS (HR 2.2, 95% CI 0.8–5.9, *p* = 0.107). Of the three patients with residual lesions after surgery, one was lost to follow-up, one died of disease progression 14 months after the diagnosis of recurrence, and one had disease progression 8 months after the last treatment but remained alive during the study period.

**Fig. 4:**
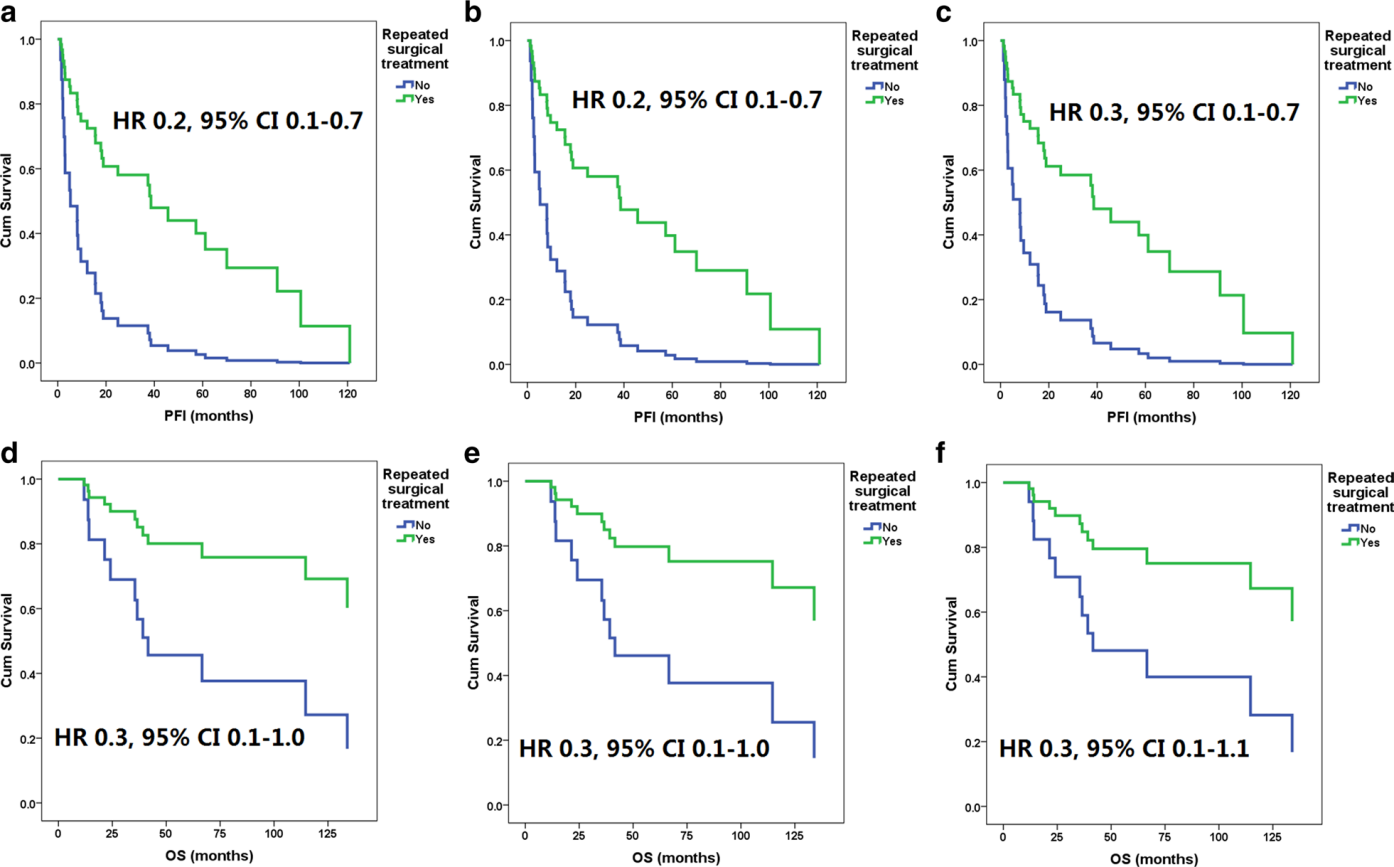
Progression-free intervals (PFI) after first recurrence in patients with and without surgical treatment adjusted with hormone treatment (**a**), chemotherapy (**b**) or radiotherapy (**c**); and overall survival (OS) after the diagnosis of first recurrence in patients with and without surgical treatment adjusted with hormone treatment (**c**), chemotherapy (**d**) or radiotherapy (**e**). *95% CI* 95% confidence interval, *HR* hazard ratio

The median OS times of the patients with and without fertility-sparing surgery were 59 (range 8–101) and 44 (3–349) months, the median OS with and without ovarian preservation were 69 (8–349) and 44 (3–229) months, and the median OS times with surgical and nonsurgical treatment were 44 (3–229) and 31 (12–349) months, respectively. No factor, including fertility-sparing surgery (Fig. 2c, HR 1.0 [95% CI 0.1–7.7], *p* = 0.977), ovarian preservation (Fig. 2d, HR 0.3 [95% CI 0.04–2.5], *p* = 0.276), and surgical treatment (HR 0.3 [95% CI 0.1–1.0], *p* = 0.052), was found to be associated with increased mortality risk. However, as shown in Fig. 4, after adjusted with hormone treatment or chemotherapy, surgical treatment had significant impact on OS (HR 0.3 and 0.3 [95% CI 0.1–1.0 and 0.1–1.0], *p* = 0.045 and 0.049, respectively). After adjusted with radiotherapy, surgical treatment still had no significant impact on OS (HR 0.3 [95% CI 0.1–1.1], *p* = 0.066).

Regarding repeated recurrent sites, in 11 and 19 patients with recurrence beyond and within the pelvic cavity at first recurrence, 6 (54.5%) and 7 (36.8%) developed new recurrence beyond the pelvic cavity, respectively (*p* = 0.287). As shown in the Additional file 1, 11 patients underwent retroperitoneal lymphadenectomy at first treatment and had no recurrences in the retroperitoneal cavity. Among 45 patients without retroperitoneal lymphadenectomy at first treatment, 4 of 45 (8.9%) and 2 of 22 (9.1%) patients had recurrences in the retroperitoneal lymph nodes at first and repeated recurrence, respectively. However, only one case had recurrence solely in the retroperitoneal cavity at first recurrence. In other cases, retroperitoneal recurrences were accompanied by recurrences in other sites.

Pathological characteristics of ER, PR expression and low mitotic activity were not associated with PFI (log-rank *p* values 0.495, 0.119 and 0.564) or OS (log-rank *p* values 0.473, 0.421 and 0.070)in the Kaplan–Meier analysis.

Hormone treatment was not associated with PFI or OS in Kaplan–Meier analysis (log-rank *p* values 0.714 and 0.717), or adjusted with ER status (*p* values 0.806 and 0.651), or PR status (*p* values 0.868 and 0.595), or surgical treatment (HR 0.9 and 0.7 [95% CI 0.4–2.1 and 0.2–2.4], *p* values 0.854 and 0.553).

Chemotherapy was not associated with PFI or OS in Kaplan–Meier analysis (log-rank *p* values 0.534 and 0.647), or adjusted with surgical treatment (HR 1.0 and 0.7 [95% CI 0.5–2.2 and 0.2–2.4], *p* values 0.806 and 0.584).

### Fertility outcomes

Among six patients requesting repeated fertility-sparing treatment, one was lost to follow-up, and the other five experienced repeated recurrence. One patient (LGE0004 in the Additional file 1) was diagnosed with a first recurrence during her cesarean section for her second live birth, when metastasis to the retroperitoneal lymph nodes was noticed. Then, she underwent repeated fertility-sparing surgeries again (resection of metastatic lymph nodes) without adjuvant therapy. However, five months after surgery, recurrence in the uterus was noticed; she accepted non-fertility-sparing surgeries and remained alive up to the end of the follow-up.

## Discussion

We report the prognosis of recurrent LGESS in a cohort of 56 patients. To our knowledge, this is the largest population to date. Compared with primary LGESS patients who had 5-year DFS and OS rates of over 90% 5-year, patients with LGESS recurrence had higher recurrence and mortality rates. Patients with fertility-sparing treatment, ovarian preservation, or a treatment modality other than repeated surgeries all had significantly higher recurrence rates. Although no factor was found to be associated with mortality risk, surgeries seemed to have marginal protective effects on OS. These findings provide a substantial basis for decision-making in the selection of treatment for LGESS recurrence.

Fertility-sparing treatment has been reported only in case reports for primary uterine LGESS [15–23], which lack evidence of safety and effectiveness. The safety of fertility-sparing treatment could be indirectly investigated by reviewing evidence of ovarian preservation. Ovarian preservation has been proven to increase the risk of LGESS recurrence in retrospective cohort studies [24–26] and in a systematic review [14]. Estrogens or tamoxifen treatment also increase the risk of recurrence [27–29], while menopause is a protective factor associated with improved PFS among patients with LGESS [30]. In addition, numerous reports have revealed the role of endometriosis in the pathogenesis of extrauterine LGESS [31, 32]. These findings all show the high risk of recurrence associated with ovarian preservation and, similarly, the high risk associated with fertility-sparing treatment. In our current study, all patients undergoing repeated fertility-sparing surgeries experienced repeated recurrence without successful conception. Based on these findings, we concluded that patients with recurrent LGESS who undergo fertility-sparing treatment have a high risk recurrence, but with few chances of conception; oophorectomy should be incorporated into the set of repeated surgeries for all recurrent LGESS patients, if applicable.

In our study, surgical treatment led to a better PFI and, possibly, a better OS for recurrent LGESS patients. Incomplete surgery and no adjuvant treatment in ESS are associated with poor DFS [6]. Several reports have confirmed that tumor size is associated with the survival outcomes of LGESS patients [5, 33, 34]. Even for patients with tumors that are difficult to resect surgically, if surgical treatment can minimize the tumor volume, sustained treatment may achieve favorable disease control [7–11]. Therefore, the principle of efforts to perform complete surgeries should also be utilized in recurrent LGESS, and surgery should be the choice of treatment for recurrent LGESS. Due to the scarcity of current evidence, a large-sample study is needed to determine the role of combination therapy consisting of surgeries and other modalities.

In our study, for patients without retroperitoneal lymphadenectomy at first treatment, approximately 10% developed retroperitoneal recurrences. The role of lymphadenectomy in LGESS is controversial. There is no sufficient indication for a systematic LND for patients with early-stage LGESS [35, 36]. A systematic LND might be necessary if enlarged lymph nodes are detected by image graphology or observation during surgery [37, 38]. In addition, lymph node metastasis does not seem to affect the excellent OS of LGESS patients [39]. Even though a high proportion (10%) of recurrent patients had recurrence in the retroperitoneal space in our study, only one patient had disease restricted in this location. These findings suggest that systematic lymphadenectomy may be eliminated in the first surgical treatment for LGESS.

In our study, we discovered that estrogen and progesterone receptor expression or mitotic activities had no significant impact on the survival outcomes of recurrent LGESS patients. There was no study on the association of mitotic activity with survival outcomes in recurrent LGESS. Low mitotic activities were associated with decreased risk of recurrence or mortality in primary diseases by some reports [25, 40, 41], but the limited sample sizes restrict the generation of their findings. The status of hormone receptors provided indication of utilization of hormone treatment. However, even for primary tumors, the effects of hormone treatment have lots of debates [30, 42–45]. In our study, although hormone treatment had no significant impact on the survival outcomes, it seemed to be able to provide survival benefits together with surgical treatment for recurrent patients.

In our study, just similar to hormone treatment, chemotherapy had no significant impact on survival outcomes, but could benefit the patients with surgical treatment. Administration of adjuvant radiotherapy or chemotherapy is not routinely used in LGESS and its role is still debated [35, 45]. Retrospective cohort study revealed that chemotherapy had no significant effect on disease-free survival of LGESS [2]. However, in the nomogram for survival of LG-ESS, chemotherapy was included as a variable in population-based analysis [33]. For the recurrent LGESS, to our knowledge, we first discovered the effectiveness of adjuvant chemotherapy for surgical treatment.

The relatively large sample size and detailed description of treatment are the strengths of our study. However, the retrospective study design is the main limitation of our study. A high rate of loss to follow-up (23.2%) after complete treatment for recurrence is another important limitation, as this restricted the generalization of our findings. National or international registry systems would provide a high-quality platform for the comprehensive analysis of rare diseases, such as recurrent LGESS.

## Conclusions

Fertility-sparing surgery or ovarian preservation treatment resulted in an inferior PFI without successful conception, and should not be offered to patients with recurrent LGESS. Surgical treatment was associated with better survival outcomes than other therapy modalities without surgeries, especially followed by adjuvant therapy of hormone treatment and/or chemotherapy, and should be the choice for recurrent LGESS. Retroperitoneal involvement occurred in approximately 10% of patients with first and repeated recurrences but seldom occurred alone, and systematic lymphadenectomy seemed to unnecessary in the primary treatment for LGESS. Pathological characteristics had no significant impact on survival outcomes.

## Supplementary Information

**Additional file 1.** Raw data of this study.

## Data Availability

All data of this study has been contained in the supplement file.

## Abbreviations

95% CI: 95% Confidence interval
DFS: Disease-free survival
HR: Hazard ratio
LGESS: Low-grade endometrial stromal sarcoma
OS: Overall survival
PFI: Progression-free interval
